# Determination of free fatty acids and volatile compounds of butter oil produced from pasteurized and unpasteurized butter at different temperatures

**DOI:** 10.1002/fsn3.4433

**Published:** 2024-08-29

**Authors:** Tekin Demir, Seval Andiç, Şehriban Oğuz

**Affiliations:** ^1^ Ziraat Bank Van Turkey; ^2^ Food Engineering Department Van Yuzuncu Yil University Van Turkey

**Keywords:** Atherogenic index, butter, butter oil, free fatty acids, volatile components

## Abstract

In this study, the effects of different raw materials, different processing temperatures, and storage temperatures on some properties of butter oil were investigated. Two different kinds of butter were produced from cream containing 40% milk fat. Both butter samples were processed into butter oil at three different temperatures (60, 90, and 120°C). Butter and butter oil samples were stored at +4°C and analyses were performed at 0, 30, and 60 days of storage. There are no significant differences between the atherogenicity index and the saturated and unsaturated fatty acid composition of butter and butter oil samples. Free fatty acid values of all samples increased during storage. Also, in all three storage periods, it was determined that free fatty acids were higher in butter samples than in butter oil samples. During storage, saturated and unsaturated free fatty acid values are generally higher in butter oil processed at 60°C than in butter oil processed at 90°C and 120°C. In total, 40 volatile compounds were detected, which included 8 ketones, each of 6 aldehydes, alcohols, acids, and hydrocarbons, 5 terpenes, and 3 esters in butter and butter oil samples. Aldehydes and ketones were generally highest in butter oil processed at 120°C.

## Graphical abstract

The most dominant saturated fatty acid is hexadecanoic acid, and unsaturated fatty acid is 9‐octadecenoic acid in butter samples. Free fatty acid values of all samples increased during storage. A total of 40 volatile components were identified in both butter and butter oil.

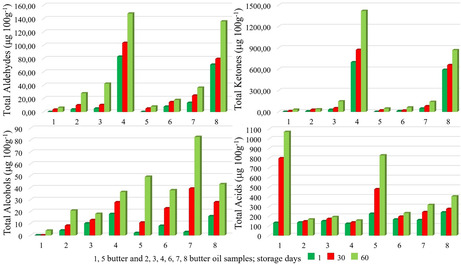

## 1 INTRODUCTION

Milk fat is an important component of milk both in terms of nutrition and economic value. It also plays a significant role in the nutritional content, structural, and sensory properties of many dairy products. Butter is a product that contains a high proportion of milk fat. Additionally, it may contain water and small amounts of other milk components. To obtain butter, a significant portion of non‐fat solids and water are removed from milk. Butter should contain a minimum of 80% milk fat and a maximum of 16% water (Turkish Food Codex, 2005). The high water content and water activity of butter make it chemically and microbiologically unstable (Fındık & Andiç, 2017). Microbiological and chemical deterioration in butter occurs in the water phase or at the water–oil interface. By removing water from the structure, butter becomes a more stable product and its shelf life is extended (Mortensen, 2011). Therefore, in many countries, especially those with hot climates and limited refrigeration facilities, butter is processed into a product with lower water activity, known as “sadeyağ” in Turkey, “ghee” or “samn” in the Middle East, “ghee” in Ethiopia and India, “roghan” in Iran, “samin” in Sudan, “samna” in Egypt, “samuli” in Uganda, and “Sary mai” in Kyrgyzstan (Chalabi et al., 2018; Gemechu & Tola, 2017; Smanalieva et al., 2022; Wadodkar et al., 2002). This product, which is known by different names in different countries, is commonly used as ghee or butter oil in the literature (Gemechu & Tola, 2017). According to the Codex Standard (CODEX STAN 280–1973), butter oil is defined as a product containing at least 99.6% milk fat (International Dairy Federation (IDF), 1997). In contrast, the Turkish Food Codex is defined as a dairy product containing at least 99% milk fat (Turkish Food Codex, 2005).

Butter oil can be produced from various raw materials such as milk, cream, and yogurt in different countries. It is mostly obtained by applying high‐temperature processing to butter. During this thermal process, phase separation occurs in the butter. Most of the buttermilk and solid‐phase components are removed from the butter. The amount and duration of heat applied in the production of butter oil can vary depending on the regions where the butter oil is produced (Demir & Andiç, 2021; Kumbhare et al., 2023). The heat treatment applied to butter leads to the denaturation of the proteins surrounding the fat globules, which allows the release of fat. Phase separation enables the removal of a significant amount of water and non‐fat solids from the butter, resulting in a product with low water activity (Das et al., 2023; Fındık & Andiç, 2017; Sserunjogi et al., 1998). The low water activity of butter oil makes it chemically and microbiologically more stable than butter.

Butter oil is produced on an industrial scale by subjecting butter to low‐temperature (60–65°C) thermal treatment and using centrifugation techniques (Özkanlı & Kaya, 2007). In small‐scale and home‐based productions, butter oil is often subjected to uncontrolled high‐temperature (110–120°C) processing (Kumbhare et al., 2023). The application of high temperatures ensures the inactivation of most of microorganisms and enzymes in butter oil, making the product microbiologically safe and preventing enzymatic hydrolysis, which is a prerequisite for autoxidation (Fındık & Andiç, 2017; Nekera et al., 2023). However, high temperatures can cause thermal oxidation of lipids and the formation of oxidation products that are harmful to health (Johnson & Decker, 2015; Wang et al., 2023). Oxidation is one of the most important quality parameters in foods and causes both a decrease in the nutritional value of foods and many sensory and structural deteriorations (Wang et al., 2023; Zhuang et al., 2022).

Studies on the subject have reported that peroxide values of butter oil obtained from butter by applying heat treatment above 100°C were higher than butter and also increased over time (Fındık & Andiç, 2017; Nekera et al., 2023). Therefore, it is important to investigate the effects of high‐temperature application on the quality characteristics of butter.

It has been reported that butter oil has several advantages over butter, such as lower water activity, microbial load, and cholesterol content (Demir & Andiç, 2021; Fındık & Andiç, 2017; Nekera et al., 2023). However, the low cholesterol content in clarified butter may also be associated with the formation of cholesterol oxidation products due to high temperature. Studies have indicated that cholesterol oxidation products are formed at temperatures above 150°C (Deng et al., 2023; Fındık & Andiç, 2017; Sieber, 2005).

The characteristics of butter and the production temperature have important effects on the nutritional value, stability, sensory, and structural properties of butter oil. Butter is not only a high‐energy source and a delicious product but also a functional food due to its essential fatty acids and components like conjugated linoleic acid. For these reasons, it is important to determine the effects of butter characteristics and production temperatures on the properties of butter oil. Additionally, it is essential to assess the extent to which free fatty acids and volatile components, which contribute to the unique butter aroma, are retained in butter oil. In addition to butter aroma substances, high production temperatures also contribute to the aroma of butter oil by causing the formation of aroma compounds (Kumbhare et al., 2023). The aroma plays a significant role in the acceptability of food products (Newton et al., 2012; Shahidi, 2001).

In some previous studies on the subject, the effects of low production temperatures (60, 70, and 80°C), and in others, the effects of high production temperatures on some properties of butter and butter oil were investigated (Fındık & Andiç, 2017; Özkanlı & Kaya, 2007; Sevmiş et al., 2020). However, there are very few studies investigating the effects of raw materials, storage times, and especially production temperatures on the free fatty acid content and volatile components of butter oil. In the production of butter oil, the process temperature was chosen as 120°C for traditional production, 60°C for industrial production, and 90°C for alternative production. In this study, the effects of pasteurization and culture addition in butter, different temperatures used in butter oil production, and storage time on the fatty acid composition, free fatty acids, and volatile components of butter and butter oil were investigated.

## 2 MATERIALS AND METHODS

**FIGURE 1:**
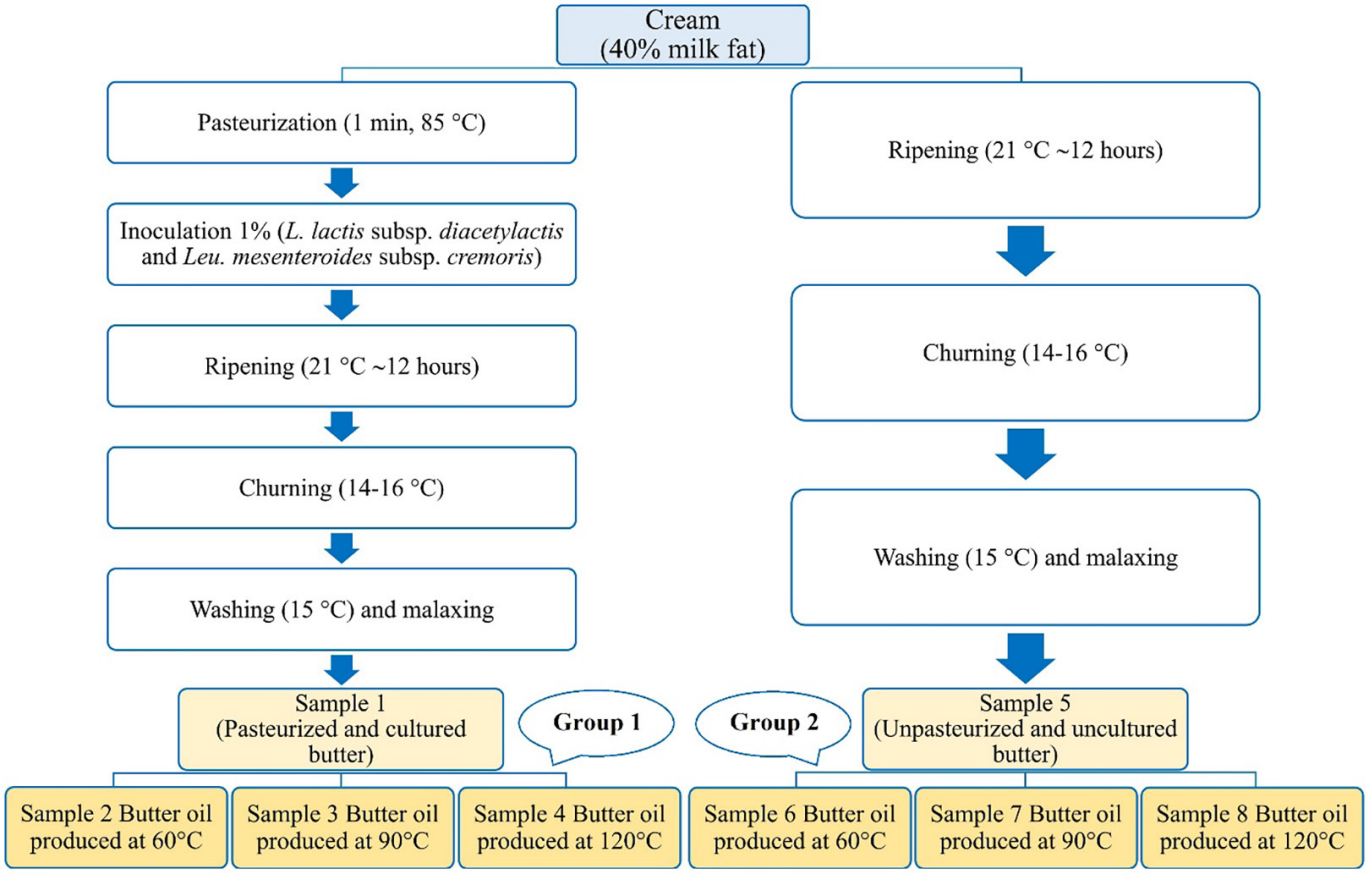
Production of butter and butter oil samples. Group 1. Pasteurized and cultured butter (sample 1) and butter oil samples produced pasteurized and cultured butter (samples 2, 3, and 4). Group 2. Unpasteurized and uncultured butter (sample 5) and butter oil samples produced unpasteurized and uncultured butter (samples 6, 7, and 8).

In the study, butter and butter oil samples produced from the cream (Baskın Süt, Hakkari, Turkey) containing 40% milk fat were used as materials. Cream used for the production of butter is divided into two parts. Part 1 of the cream was pasteurized at 85°C for 1 minute and after cooling to 21°C was inoculated with a starter culture (Danisco, Germany) (*Lactococcus lactis* subsp. *lactis* bv. *diacetylactis* and *Leuconostoc mesenteroides* subsp. *cremoris*) at 1%. The second part cream was not pasteurized as the control group. Pasteurized and unpasteurized creams were matured until their pH reached 5.0. The ripening process was done at 21°C and took about 12 hours. The creams, whose maturation process was completed, were churned at approximately 15°C. The oil granules were washed with cold water at 15°C and malaxing was performed. Butter produced from both cream was divided into four groups and group 1 was used as butter sample. The other three groups of samples were processed into butter oil at 60, 90, and 120°C. The samples of butter and butter oil were stored at +4°C for 60 days and analyses were performed on days 0, 30, and 60 of storage. Production of butter and butter oil samples are given in Figure 1.

### 2.1 Determination of total fatty acids by gas chromatography

The fat in the samples was extracted using Folch method (Folch et al., 1957). Fatty acid methyl esters (FAMEs) are derivatives of fatty acids that are commonly used in gas chromatography (GC) analysis to determine the fatty acid composition of lipid samples (García‐González et al., 2018). The fatty acid composition of butter and butter oil samples was determined using the International Union of Pure and Applied Chemistry (IUPAC) method 2.301 for the preparation of the FAMEs (with 2 N KOH in methanol). The FAMEs were analyzed by GC (Agilent 6890 N, USA) with an HP‐Innowax capillary column (60 m × 0.25 μm × 0.2 mm ID) following the IUPAC method 2.302 for gas chromatography analysis (IUPAC, 1992). The column temperature was increased from 100°C to 200°C at a rate of 5°C/min. A flame ionization detector (FID) was used, with hydrogen and dry air at 240°C. Helium gas was used as the carrier gas at a flow rate of 0.9 mL/min, and the injection block temperature was set to 230°C. The Supelco FAME 37 component mix (Supelco, Bellefonte, PA, USA) served as the standard. Chromatogram evaluation was performed using Chem Station software (A.10.02, Agilent) and the NIST database. FAME percentages were quantified based on their relative peak areas.

### 2.2 Calculation of atherogenic index

Nutritional quality of the lipid fractions was evaluated using the atherogenic index (AI) (Ulbricht & Southgate, 1991).

$$\mathrm{IA}=C12:0+\left(4\times C14:0\right)+C16:0/\sum \mathrm{UFA}.$$

C12:0 = Dodecanoic acid (Lauric acid). C14:0 = Tetradecanoic acid (Myristic acid). C16:0 = Hexadecanoic acid (Palmitic acid). ∑UFA = Total unsaturated fatty acids.

### 2.3 Determination of free fatty acids by gas chromatography

For the analysis of free fatty acids, 1 g of sample and 3 g of anhydrous sodium sulfate were mixed. To this mixture, 0.3 mL of sulfuric acid (2.5 mol/L) and 1 mL of internal standard (0.5 mg/mL C5:0 and 0.5 mg/mL C13:0) were added and the mixture was extracted three times with 3 mL of ether/heptane (1:1). The solvent was passed two times through the aminopropyl column conditioned with 10 mL of heptane to retain the free fatty acids in the column. After removing neutral lipids from the column with 10 mL ether/heptane mixture (1:1), free fatty acids (FFA) were eluted with diethyl ether containing 0.3 mL/mL formic acid and injected into the gas chromatography (QP 2010 Ultra SHIMADZU brand GC–MS) device (De Jong & Badings, 1990). The identification of free fatty acids in butter and butter oil samples was accomplished by comparing the retention times of sample FFAs with those of FFA standards (Sigma‐Aldrich, Buchs, Switzerland) under identical conditions. The injector temperature was 250°C in split mode (1:10). The initial oven temperature (60°C) increased to 240°C at 10°C/min and was maintained for 45 min. Thermo Scientific FFA Capillary column (260 × 298P L: 30 m × 0.530 mm, film thickness: 0.25 μm, Thermo Scientific Cheshire, UK) was used in the separation of FFAs. Helium was used as the carrier gas at a flow rate of 2.6 mL/min. The mass spectrometer (MS) transfer line temperature was 240°C and the ion source temperature was 230°C. The scan range was adjusted from 40 to 450 amu.

### 2.4 Determination of volatile components

Four grams of butter and butter oil were replaced in a 40 mL screw‐top headspace glass vial with secure seal (Santa Clara, CA, USA) and 10 μL of 5‐methyl 2‐hexanone was added as internal standard (IS). After 5 min equilibration at 40°C with constant stirring using a digital hot plate (stirred at 1000 rpm), the A 2 cm solid‐phase microextraction (SPME) fiber (50/30 μm divinylbenzene/carboxen/polydimethylsiloxane, Supelco Co., Bellefonte, PA, USA) was exposed for 30 min to the headspace for volatile extraction at the same temperature. After the extraction, the volatiles were desorbed in the injection port of a Shimadzu gas chromatography–mass spectrometry (GC–MS)‐QP2010 (Shimadzu Corporation, Kyoto, Japan). The injector temperature was set at 250°C and splitless injection mode was used for 5 min. The samples were analyzed on a Rtx‐5MS column (30 m × 0.25 mm × 0.25 μm; Restek Corp., Bellefonte, PA, USA). Helium was used as the carrier gas at a constant flow rate of 1.5 mL/min. The oven temperature was programmed at 35°C for a hold of 5 min, increased to 75°C at a rate of 8°C/min, then increased to 220°C at a rate of 40°C/min, and held at the final temperature for 20 min. The mass spectrometer (MS) transfer line temperature was 250°C and the ion source temperature was 200°C. The scan range was adjusted from 40 to 450 amu. Internal standard peak area ratio was used to calculate the amounts of each volatile compound. Peak identification of volatiles was based on comparison of the MS spectra with the W9N11 and FFNSC1.2 mass spectra libraries. An n‐alkane series (C5–C27) was used to calculate the linear retention index (RI) of each volatile compound (Prosen & Zupančič‐Kralj, 1999).

### 2.5 Statistical analyses

Volatile component and free fatty acids values obtained in the study were subjected to analysis of variance by SPSS software 2000. Duncan's multiple‐comparison test (significance *p* < .05) was used to determine whether the differences between the means were significant. All analyses were performed in three parallels and the results were given as mean ± standard deviation (SD).

## 3 RESULTS AND DISCUSSION

### 3.1 Fatty acids and atherogenic index

The fatty acid analysis of butter and butter oil samples was conducted only at the beginning of storage, assuming that there would be no significant changes over time. The fatty acid compositions of butter (cultured and uncultured) and butter oil (derived from cultured and uncultured) were found to have no statistically significant differences. The total saturated and unsaturated fatty acid compositions of the samples are provided in Table 1.

**TABLE 1:** Total fatty acid values of butter and butter oil samples (g/100 g) (values are means ± SD, *n* = 3).

Sample no	Total saturated fatty acids (g/100 g)	Total unsaturated fatty acids (g/100 g)	Atherogenicity index
1	69.22 ± 4.10	30.78 ± 0.81	2.77 ± 7.34
2	68.03 ± 6.00	31.97 ± 1.21	2.80 ± 14.70
3	69.56 ± 3.43	30.44 ± 4.03	2.91 ± 5.29
4	70.01 ± 6.12	29.99 ± 2.67	2.73 ± 3.99
5	69.18 ± 5.56	30.82 ± 5.00	2.80 ± 4.61
6	69.00 ± 2.22	31.00 ± 0.37	2.72 ± 11.02
7	70.04 ± 0.67	29.96 ± 6.98	2.56 ± 2.09
8	69.56 ± 1.38	30.44 ± 0.99	2.87 ± 6.31

In addition to their nutritional values, fats in the composition of foods can be risk factors or have a protective effect for some diseases, depending on the type and number of fatty acids (chain length, degree of saturation, and geometric isomer type) in their structure. Total cholesterol and saturated fatty acids are considered risk factors for coronary heart disease. On the other hand, specific fatty acids, like omega‐3 fatty acids, are known for their potential health benefits, including cardiovascular protection (Bo et al., 2018; Schacky & Harris, 2007; Siri‐Tarino et al., 2010; Virtanen et al., 2014).

Various methods are available for determining the quality of fats in foods. The atherogenic index is a measure that indicates the relationship between saturated and unsaturated fatty acids present in foods (Chen & Liu, 2020; Javardi et al., 2020). Saturated fatty acids, especially C12:0 (lauric acid), C14:0 (myristic acid), and C16:0 (palmitic acid), are considered proatherogenic because they support the adhesion of lipids to circulation and immune system cells. Such fatty acids can increase low‐density lipoprotein (LDL – bad cholesterol) levels in the blood and contribute to the development of cardiovascular diseases such as atherosclerosis. On the other hand, unsaturated fatty acids are considered antiatherogenic due to their effects in preventing plaque accumulation and lowering cholesterol, phospholipids, and esterified fatty acids levels (Balta et al., 2021; Majdalawieh et al., 2020; Ulbricht & Southgate, 1991).

In our study, similar to the findings of the fatty acid composition of butter and butter oil samples, there was no statistically significant difference between the AI values. Ulbricht and Southgate reported an AI of 2.03 for milk, cheese, and butter. AI values were reported for Holstein cow's milk 2.23–2.90, for Jersey cow's milk 3.98–4.66, for sheep milk yogurt 0.65–1.68, and for cow milk yogurt 0.39–1.84 (Ahmad et al., 2020; Salles et al., 2019; Vargas‐Bello‐Pérez et al., 2015). The variations in AI values are mainly due to differences in animal species and diet.

### 3.2 Free fatty acids

The values of free fatty acids in butter and butter oil samples are given in Tables 2 and 3, respectively, and in all samples, the most dominant saturated fatty acid is hexadecanoic acid (palmitic acid), while the most dominant unsaturated fatty acid is 9‐octadecenoic acid (oleic acid). Throughout the storage period, the free fatty acid values of butter samples in both groups are higher than those of butter oil samples. Similar results have also been reported by Fındık and Andiç (2017) and Demir and Andiç (2021). In butter oil production, a portion of the free fatty acids present in butter separates from the structure along with water and non‐fat solids. However, at the beginning of storage, the differences between butter and butter oil in terms of free fatty acids are more pronounced, especially in short‐chain and unsaturated fatty acids. This is because the solubility of fatty acids in water increases as the chain length decreases and the degree of unsaturation increases (Nawar, 1996).

**TABLE 2:** Saturated free fatty acid values (mg/kg) of butter and butter oil samples (values are means ± SD, *n* = 3).

Sample no	Storage time (days)					
	0	30	60	0	30	60
	Butanoic acid (C4:0)			Hexanoic acid (C6:0)		
1	26.45 ± 0.69 ^Ac^	170.15 ± 1.54 ^Bb^	335.06 ± 1.51 ^Ba^	24.24 ± 1.49 ^Ac^	145.86 ± 2.38 ^Bb^	351.00 ± 0.03 ^Aa^
2	8.48 ± 0.03 ^Cb^	8.55 ± 0.07 ^Cb^	11.09 ± 0.89 ^Ea^	17.70 ± 0.92 ^Cb^	18.52 ± 1.30 ^Dba^	20.03 ± 0.05 ^Ca^
3	8.96 ± 1.31 ^CBc^	11.73 ± 1.91 ^Cba^	12.63 ± 0.06 ^Ca^	18.48 ± 1.90 ^CBb^	24.05 ± 1.05 ^Ca^	24.54 ± 1.05 ^Ca^
4	8.57 ± 0.04 ^Cc^	9.23 ± 0.04 ^Cb^	10.29 ± 0.02 ^Ca^	12.67 ± 0.97 ^Da^	13.52 ± 0.99 ^Ea^	14.08 ± 1.02 ^Ca^
5	26.89 ± 1.05 ^Ac^	206.97 ± 4.43 ^Ab^	367.44 ± 6.77 ^Aa^	25.56 ± 1.97 ^Ac^	183.14 ± 4.00 ^Ab^	231.86 ± 17.63 ^Ba^
6	9.41 ± 0.05 ^CBb^	9.47 ± 0.07 ^Cb^	10.44 ± 0.10 ^Ca^	18.76 ± 1.01 ^Cb^	21.52 ± 0.98 ^DCa^	23.00 ± 1.08 ^Ca^
7	10.11 ± 0.04 ^Bc^	10.96 ± 0.03 ^Cb^	13.65 ± 0.72 ^Ca^	20.04 ± 0.07 ^CBb^	21.74 ± 0.03 ^DCba^	21.87 ± 1.11 ^Ca^
8	8.56 ± 0.04 ^Cb^	9.24 ± 0.04 ^Cb^	10.55 ± 0.97 ^Ca^	20.70 ± 0.96 ^Bb^	22.68 ± 0.75 ^Cba^	23.24 ± 1.28 ^Ca^
	Octanoic Acid (C8:0)			Decanoic Acid (C10:0)		
1	54.48 ± 3.59 ^Ac^	277.91 ± 16.79 ^Ab^	726.49 ± 12.27 ^Aa^	104.78 ± 5.61 ^Ac^	504.73 ± 7.61 ^Bb^	1445.34 ± 61.63 ^Aa^
2	21.25 ± 2.03 ^Bc^	23.20 ± 1.67 ^Cb^	26.53 ± 3.65 ^Ca^	77.42 ± 3.39 ^Bc^	112.96 ± 5.51 ^Cb^	131.18 ± 1.62 ^Ca^
3	21.50 ± 0.86 ^Ba^	25.04 ± 3.64 ^Ca^	25.80 ± 2.40 ^Ca^	52.61 ± 5.71 ^Da^	58.82 ± 6.93 ^Da^	62.51 ± 4.26 ^Da^
4	20.44 ± 0.56 ^Bc^	22.61 ± 0.77 ^Cb^	24.33 ± 0.50 ^Ca^	52.45 ± 1.26 ^Da^	53.94 ± 3.63 ^Da^	54.62 ± 3.49 ^Da^
5	52.84 ± 1.96 ^Ac^	265.48 ± 6.90 ^Bb^	428.13 ± 2.45 ^Ba^	106.98 ± 5.97 ^Ac^	539.57 ± 48.75 ^Ab^	1049.08 ± 68.54 ^Ba^
6	20.95 ± 3.30 ^Bb^	25.22 ± 2.79 ^Cba^	27.79 ± 3.15 ^Ca^	103.02 ± 0.05 ^Cc^	122.28 ± 3.25 ^Cb^	130.14 ± 2.56 ^Ca^
7	23.40 ± 2.35 ^Ba^	24.87 ± 0.97 ^Ca^	25.88 ± 1.57 ^Ca^	51.77 ± 2.33 ^Db^	53.56 ± 2.46 ^Dba^	58.56 ± 3.03 ^Da^
8	20.91 ± 2.86 ^Ba^	25.71 ± 4.55 ^Ca^	25.01 ± 3.93 ^Ca^	51.31 ± 3.60 ^Db^	54.41 ± 1.45 ^Db^	59.74 ± 1.09 ^Da^
	Dodecanoic Acid (C12:0)			Tetradecanoic Acid (C14:0)		
1	162.18 ± 0.96 ^Bc^	174.30 ± 3.00 ^Ab^	180.23 ± 1.02 ^Aa^	256.30 ± 1.18 ^Bc^	347.95 ± 17.83 ^Ab^	560.22 ± 28.82 ^Aa^
2	118.74 ± 1.01 ^Cc^	128.83 ± 1.50 ^Bb^	141.11 ± 1.37 ^Ca^	250.63 ± 4.82 ^CBc^	283.60 ± 7.73 ^Bb^	308.78 ± 8.10 ^Ca^
3	116.52 ± 5.81 ^Ca^	123.70 ± 2.45 ^Ca^	124.08 ± 4.01 ^Da^	242.10 ± 2.51 ^CDb^	246.04 ± 5.03 ^DCb^	310.31 ± 8.20 ^Ca^
4	118.29 ± 0.73 ^Cb^	122.19 ± 0.94 ^Ca^	123.37 ± 2.90 ^Da^	241.11 ± 1.02 ^CDb^	271.76 ± 6.58 ^Ba^	276.27 ± 9.38 ^Da^
5	169.00 ± 0.04 ^Ab^	171.65 ± 1.49 ^Aa^	172.99 ± 1.46 ^Ba^	269.55 ± 9.23 ^Ab^	273.78 ± 2.24 ^Bb^	480.30 ± 0.98 ^Ba^
6	119.01 ± 1.35 ^Cc^	128.05 ± 2.77 ^Bb^	142.11 ± 4.45 ^Ca^	211.03 ± 3.78 ^Fb^	231.46 ± 11.65 ^Da^	234.22 ± 4.32 ^Ea^
7	118.36 ± 1.30 ^Cb^	121.12 ± 1.81 ^Cb^	125.40 ± 1.79 ^Da^	229.77 ± 1.99 ^Ea^	231.81 ± 8.35 ^Da^	239.05 ± 9.98 ^Ea^
8	118.29 ± 0.82 ^Cc^	121.83 ± 1.21 ^Cb^	124.97 ± 1.12 ^Da^	238.65 ± 10.03 ^EDb^	249.00 ± 4.64 ^Cb^	267.45 ± 4.49 ^Da^
	Hexadecanoic Acid (C16:0)			Octadecanoic Acid (C18:0)		
1	1508.96 ± 87.50 ^Bc^	1678.13 ± 0.02 ^Bb^	3379.77 ± 0.06 ^Aa^	593.77 ± 5.84 ^Ac^	677.45 ± 16.01 ^Ab^	893.72 ± 9.03 ^Aa^
2	1228.93 ± 0.06 ^Cc^	1266.00 ± 0.04 ^Cb^	1654.28 ± 0.09 ^Ca^	404.53 ± 0.04 ^Bc^	406.95 ± 0.04 ^Cb^	611.62 ± 0.03 ^Ca^
3	1166.91 ± 56.37 ^DCb^	1237.12 ± 96.18 ^DCb^	1524.74 ± 46.77 ^Da^	338.36 ± 17.42 ^Cc^	366.29 ± 5.90 ^Eb^	525.98 ± 0.05 ^Da^
4	1150.44 ± 0.04 ^Dc^	1181.19 ± 0.06 ^Db^	1460.99 ± 0.06 ^Ea^	310.30 ± 0.86 ^Dc^	387.71 ± 0.11 ^Db^	400.08 ± 0.06 ^Ea^
5	1692.28 ± 0.04 ^Ac^	1986.81 ± 0.04 ^Ab^	2797.03 ± 0.07 ^Ba^	576.84 ± 24.36 ^Ac^	655.53 ± 0.05 ^Bb^	816.89 ± 0.04 ^Ba^
6	874.75 ± 0.06 ^Ec^	922.70 ± 0.04 ^Eb^	924.31 ± 0.03 ^Ha^	307.97 ± 15.79 ^Db^	318.85 ± 10.32 ^Gb^	350.51 ± 5.05 ^Ga^
7	883.69 ± 0.05 ^Ec^	922.85 ± 0.04 ^Eb^	993.90 ± 0.04 ^Fa^	305.55 ± 0.05 ^Dc^	335.62 ± 0.70 ^Fb^	362.21 ± 0.04 ^Fa^
8	873.50 ± 1.39 ^Ec^	886.66 ± 0.05 ^Eb^	958.76 ± 0.04 ^Ga^	316.48 ± 1.20 ^Dc^	332.11 ± 0.93 ^Fb^	334.65 ± 0.04 ^Ha^

*Note*: ^A–H^Different uppercase letters within a column and free fatty acid indicate significant differences between butter and butter oil samples according to Duncan's multiple‐range test (*p* < .05). ^a–c^Different lowercase letters within a row (sample) indicate significant differences between storage periods according to Duncan's multiple‐range test (*p* < .05).

**TABLE 3:** Unsaturated free fatty acid values (mg/kg) of butter and butter oil samples (values are means ± SD, *n* = 3).

Free fatty acids	Sample no	Storage time (days)		
		1	30	60
9‐Octadecenoic Acid (C18:1)	1	1838.59 ± 0.62 ^Ac^	2430.07 ± 1.63 ^Ab^	3051.30 ± 118.55 ^Aa^
	2	724.47 ± 12.74 ^Bb^	806.32 ± 9.25 ^DCb^	1094.97 ± 94.30 ^Ca^
	3	677.95 ± 14.04 ^Bc^	828.31 ± 45.95 ^DCb^	988.06 ± 13.48 ^Ca^
	4	645.84 ± 32.19 ^Bc^	738.27 ± 42.60 ^Db^	980.89 ± 32.61 ^Ca^
	5	1780.23 ± 130.57 ^Ac^	2183.99 ± 103.38 ^Bb^	2810.70 ± 102.48 ^Ba^
	6	727.76 ± 25.07 ^Bb^	802.56 ± 77.44 ^DCb^	1040.74 ± 75.68 ^Ea^
	7	707.11 ± 15.97 ^Bb^	856.32 ± 55.00 ^Cba^	994.20 ± 117.36 ^Ca^
	8	657.07 ± 53.28 ^Bb^	741.77 ± 64.19 ^Db^	969.93 ± 116.22 ^Ca^
9,12‐Octadecadienoic Acid (C18:2)	1	103.39 ± 10.03 ^Ac^	170.08 ± 3.35 ^Ab^	197.60 ± 19.49 ^Aa^
	2	76.76 ± 11.19 ^CBb^	85.69 ± 4.06 ^Cb^	105.54 ± 11.72 ^Ba^
	3	84.32 ± 5.43 ^Bb^	90.66 ± 2.72 ^Cb^	103.96 ± 5.50 ^Ba^
	4	80.70 ± 8.30 ^CBb^	87.05 ± 5.22 ^Cb^	102.52 ± 4.22 ^Ba^
	5	100.67 ± 7.11 ^Ac^	151.20 ± 21.42 ^Bb^	190.96 ± 11.34 ^Aa^
	6	74.67 ± 4.75 ^CBb^	83.41 ± 4.20 ^Cb^	102.61 ± 9.89 ^Ba^
	7	71.40 ± 3.23 ^CBb^	91.04 ± 9.66 ^Cba^	110.52 ± 19.05 ^Ba^
	8	67.56 ± 6.97 ^Cb^	84.46 ± 6.03 ^Cb^	105.49 ± 12.66 ^Ba^
6,9,12‐Octadecatrienoic Acid (C18:3)	1	126.85 ± 6.28 ^Ac^	165.76 ± 11.24 ^Ab^	245.33 ± 16.84 ^Aa^
	2	41.31 ± 2.20 ^Bb^	47.57 ± 2.59 ^Ca^	50.62 ± 1.76 ^Ca^
	3	35.81 ± 4.94 ^CBb^	42.15 ± 3.66 ^DCba^	47.51 ± 4.98 ^DCa^
	4	22.63 ± 1.95 ^Eb^	40.64 ± 1.86 ^DCEa^	43.13 ± 2.12 ^DCa^
	5	125.99 ± 4.49 ^Ac^	144.16 ± 4.63 ^Bb^	227.74 ± 8.20 ^Ba^
	6	28.35 ± 0.50 ^EDc^	32.34 ± 0.58 ^Eb^	34.38 ± 1.29 ^Da^
	7	31.29 ± 0.78 ^CDa^	35.14 ± 3.13 ^DEa^	35.22 ± 1.73 ^Da^
	8	31.62 ± 1.10 ^CDa^	32.95 ± 0.05 ^Ea^	35.92 ± 4.00 ^Da^

*Note*: ^A–E^Different uppercase letters within a column and free fatty acid indicate significant differences between butter and butter oil samples according to Duncan's multiple‐range test (*p* < .05). ^a–c^Different lowercase letters within a row (sample) indicate significant differences between storage periods according to Duncan's multiple‐range test (*p* < .05).

Throughout the storage period, the free fatty acid values of all samples have shown an increase. However, this increase in free fatty acid values is higher in butter samples compared to butter oil samples. Previous studies have reported that butter oil has lower acidity and water activity values compared to butter (Fındık & Andiç, 2017). This situation limits chemical hydrolysis in butter oil. In addition, the heat treatment applied during the production of butter oil causes inactivation of lipase enzymes and limits the formation of enzymatic hydrolysis (Nawar, 1996).

Limited presence of free fatty acids in certain cheeses contributes to the formation of desired aroma. However, the presence of free fatty acids in both butter and clarified butter is undesirable. Short‐chain fatty acids can cause aroma defects, while unsaturated fatty acids can lead to oxidation, resulting in the deterioration of product quality (Frankel, 2015; Nawar, 1996).

### 3.3 Volatile components

As can be seen from Tables 4, 5, 6, 7, 8, a total of 40 volatile compounds were identified in the samples of butter and butter oil, including 8 ketones, 5 terpenes, 3 esters, and 6 each of aldehydes, alcohols, acids, and hydrocarbons.

**TABLE 4:** Volatile aldehyde values (μg/100 g) of butter and butter oil samples (values are means ± SD, *n* = 3).

Sample no	Storage time (days)							
	RI	0	30	60	RI	0	30	60
	3‐Methylbutanal				Pentanal			
1	675.78	0.00 ^Aa^	0.00 ^Ea^	0.00 ^Da^	700.35	0.00 ^Cb^	0.00 ^Cb^	0.33 ± 0.04 ^Ca^
2		0.00 ^Ab^	5.51 ± 0.05 ^Ba^	0.00 ^Db^		0.00 ^Cb^	0.00 ^Cb^	7.37 ± 0.04 ^Ba^
3		0.00 ^Ac^	3.32 ± 0.03 ^Db^	6.40 ± 0.05 ^Ba^		0.00 ^Bb^	0.00 ^Cb^	6.68 ± 0.04 ^Ba^
4		0.00 ^Aa^	0.00 ^Ea^	0.00 ^Da^		13.28 ± 0.03 ^Ac^	22.92 ± 0.05 ^Ab^	37.44 ± 1.91 ^Aa^
5		0.00 ^Aa^	0.00 ^Ea^	0.00 ^Da^		0.00 ^Ca^	0.00 ^Ca^	0.00 ^Ca^
6		0.00 ^Ac^	3.82 ± 0.06 ^Cb^	4.36 ± 0.06 ^Ca^		0.00 ^Ca^	0.00 ^Ca^	0.00 ^Ca^
7		0.00 ^Ac^	6.65 ± 0.02 ^Ab^	7.92 ± 0.07 ^Aa^		0.00 ^Ca^	0.00 ^Ca^	0.00 ^Ca^
8		0.00 ^Aa^	0.00 ^Ea^	0.00 ^Da^		10.73 ± 0.06 ^Bc^	11.32 ± 0.07 ^Bb^	37.78 ± 0.05 ^Aa^
	Hexanal				Heptanal			
1	801.35	0.00 ^Ea^	0.00 ^Ea^	0.00 ^Fa^	902.11	0.00 ^Gc^	2.41 ± 0.08 ^Hb^	4.19 ± 0.09 ^Ha^
2		0.00 ^Eb^	0.00 ^Eb^	8.53 ± 0.03 ^Da^		3.75 ± 0.06 ^Eb^	3.76 ± 0.10 ^Fb^	8.42 ± 0.07 ^Ea^
3		0.00 ^Eb^	0.00 ^Eb^	16.25 ± 0.06 ^Ca^		4.20 ± 0.06 ^Cc^	5.78 ± 0.05 ^Db^	11.54 ± 0.07 ^Ca^
4		18.51 ± 0.04 ^Ac^	23.20 ± 0.08 ^Ab^	41.65 ± 0.07 ^Aa^		35.34 ± 0.08 ^Bc^	38.99 ± 0.06 ^Ab^	49.32 ± 0.06 ^Ba^
5		0.00 ^Ea^	0.00 ^Ea^	0.00 ^Fa^		0.00 ^Gc^	5.07 ± 0.06 ^Eb^	8.12 ± 0.05 ^Fa^
6		5.16 ± 0.07 ^Dc^	7.01 ± 0.06 ^Db^	7.16 ± 0.07 ^Ea^		2.10 ± 0.04 ^Fc^	2.67 ± 0.05 ^Gb^	4.61 ± 0.09 ^Ga^
7		7.76 ± 0.07 ^Cc^	8.28 ± 0.09 ^Cb^	8.56 ± 0.06 ^Da^		5.15 ± 0.03 ^Cc^	5.96 ± 0.05 ^Cb^	10.36 ± 0.09 ^Da^
8		10.77 ± 0.13 ^Bc^	15.25 ± 0.06 ^Bb^	16.57 ± 0.12 ^Ba^		35.94 ± 0.04 ^Ac^	37.56 ± 0.07 ^Bb^	55.65 ± 0.13 ^Aa^
	Octanal				Nonanal			
1	1002.86	0.00 ^Ca^	0.00 ^Ca^	0.00 ^Ca^	1104.39	0.00 ^Eb^	1.04 ± 0.07 ^Fb^	1.80 ± 0.08 ^Ea^
2		0.00 ^Ca^	0.00 ^Ca^	0.00 ^Ca^		0.00 ^Ec^	0.73 ± 0.02 ^Gb^	3.68 ± 0.04 ^Da^
3		0.00 ^Ca^	0.00 ^Ca^	0.00 ^Ca^		0.91 ± 0.09 ^Cc^	1.31 ± 0.09 ^Db^	1.59 ± 0.07 ^Ea^
4		4.14 ± 0.07 ^Ab^	5.24 ± 0.04 ^Aa^	5.40 ± 0.13 ^Ba^		11.67 ± 0.13 ^Ac^	13.27 ± 0.08 ^Ab^	13.90 ± 0.05 ^Ba^
5		0.00 ^Ca^	0.00 ^Ca^	0.00 ^Ca^		0.00 ^Ea^	0.00 ^Ha^	0.00 ^Fa^
6		0.00 ^Ca^	0.00 ^Ca^	0.00 ^Ca^		0.71 ± 0.05 ^Dc^	1.17 ± 0.05 ^Eb^	1.82 ± 0.03 ^Ea^
7		0.00 ^Ca^	0.00 ^Ca^	0.00 ^Ca^		0.74 ± 0.06 ^Dc^	3.60 ± 0.06 ^Cb^	9.51 ± 0.60 ^Ca^
8		3.68 ± 0.06 ^Bc^	4.48 ± 0.05 ^Bb^	6.93 ± 0.07 ^Aa^		9.87 ± 0.03 ^Bc^	11.00 ± 0.02 ^Bb^	18.97 ± 0.05 ^Aa^

*Note*: ^A–H^Different uppercase letters within a column and aldehyde indicate significant differences between butter and butter oil samples according to Duncan's multiple‐range test (*p* < .05). ^a–c^Different lowercase letters within a row (sample) indicate significant differences between storage periods according to Duncan's multiple‐range test (*p* < .05).

Aldehydes are the main group of compounds typically originating from the thermal oxidation of polyunsaturated triacylglycerols and play a significant role in creating the unique flavor of butter and other dairy products (Jerković et al., 2007; Shahidi, 2001; Zhang et al., 2012). The aldehydes detected in butter and butter oil include 3‐methylbutanal, pentanal, hexanal, heptanal, octanal, and nonanal (Table 4). Volatile aldehyde values showed fluctuating changes in all samples, with the highest values obtained from butter oil samples processed at 120°C.

3‐Methylbutanal was only detected in butter oil samples processed at 60°C and 90°C. The pentanal detected on days 0, 30, and 60 of storage in butter oils processed at 120°C increased significantly (*p* < .05) at the end of storage. Wadodkar et al. (2002) also reported the presence of pentanal in butter samples and its contribution to the aroma of clarified butter.

The highest hexanal values in both groups were found in butter oil samples produced at 120°C (Table 3). Andrewes (2012) reported similar results for ghee produced using direct cream, cream butter, or prestratification methods and stored at 60°C. Gundogdu et al. (2020) reported an increase in hexanal values in butter samples produced from cream and stored for 60 days.

**FIGURE 2:**
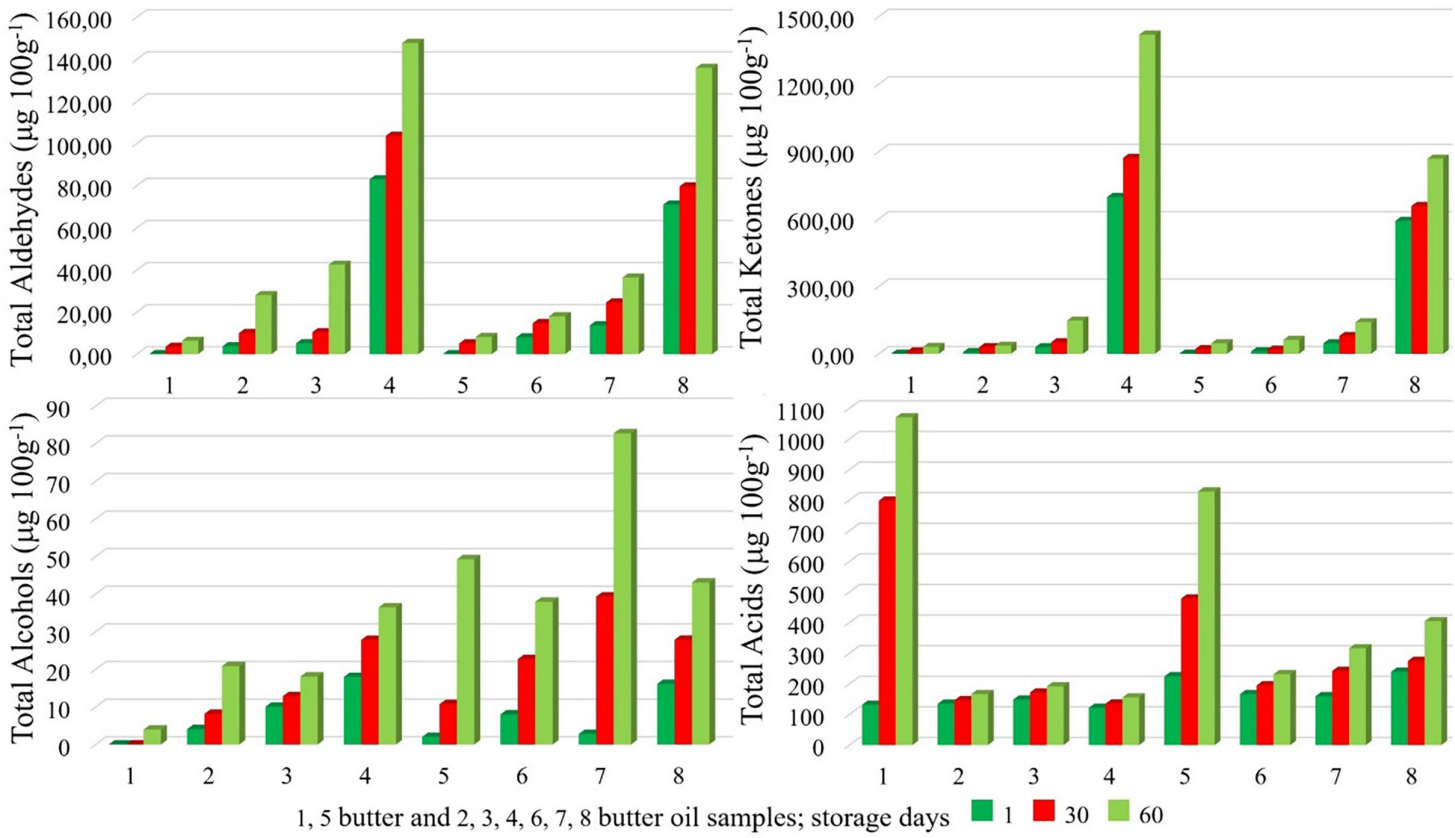
Changes in total volatile aldehyde, ketone, alcohol, and acid amounts in butter and butter oil samples during 60‐day storage.

Among all samples, the highest total aldehyde values were observed in butter oil samples produced at 120°C (Figure 2). The significant increase in aldehydes in butter samples depending on production temperature and storage time suggests the occurrence of thermal and auto‐oxidation. Aldehydes and ketones are the main volatile compounds formed during auto‐oxidation and, when their concentrations are high enough, they can lead to painty, fatty, metallic, papery, and candle‐like flavors in foods (Lindsay, 1996; Newton et al., 2012).

Eight ketones were identified in both butter and butter oil samples, with 2‐heptanone being the most dominant ketone (Table 5). Similar to our findings, Lee et al. (1991) reported 2‐heptanone as the most dominant ketone for butter in their study. 3‐Methyl‐2‐pentanone, 2‐pentanone, and 2‐decanone were only detected in butter oil samples throughout all analysis periods.

**TABLE 5:** Volatile ketone values (μg/100 g) of butter and butter oil samples (values are means ± SD, *n* = 3).

Sample no	Storage time (days)							
	RI	0	30	60	RI	0	30	60
	2‐Propanone				3‐Methyl‐2‐pentanone			
1	558.49	0.00 ^Ab^	0.00 ^Bb^	16.10 ± 0.15 ^Ca^	651.05	0.00 ^Ba^	0.00 ^Ca^	0.00 ^Ca^
2		0.00 ^Aa^	0.00 ^Ba^	0.00 ^Ea^		0.00 ^Ba^	0.00 ^Ca^	0.00 ^Ca^
3		0.00 ^Ab^	0.00 ^Bb^	80.00 ± 0.14 ^Aa^		0.00 ^Ba^	0.00 ^Ca^	0.00 ^Ca^
4		0.00 ^Aa^	0.00 ^Ba^	0.00 _Ea_		0.00 ^Bc^	8.72 ± 0.04 ^Bb^	29.86 ± 0.09 ^Ba^
5		0.00 ^Ac^	2.62 ± 0.05 ^Ab^	10.10 ± 0.06 ^Da^		0.00 ^Ba^	0.00 ^Ca^	0.00 ^Ca^
6		0.00 ^Ab^	0.00 ^Bb^	28.03 ± 2.11 ^Ba^		0.00 ^Ba^	0.00 ^Ca^	0.00 ^Ca^
7		0.00 ^Aa^	0.00 ^Ba^	0.00 ^Ea^		0.00 ^Ba^	0.00 ^Ca^	0.00 ^Ca^
8		0.00 ^Aa^	0.00 ^Ba^	0.00 ^Ea^		15.99 ± 0.06 ^Ac^	18.59 ± 0.10 ^Ab^	35.62 ± 0.04 ^Aa^
	2‐pentanone				2‐heptanone			
1	689.38	0.00 ^Ba^	0.00 ^Ea^	0.00 ^Ea^	941.28	0.00 ^Gc^	10.23 ± 0.08 ^Hb^	10.84 ± 0.11 ^Ha^
2		0.00 ^B^c	16.11 ± 0.05 ^Cb^	18.04 ± 0.06 ^Ca^		7.33 ± 0.11 ^Fc^	12.85 ± 0.11 ^Gb^	15.81 ± 0.12 ^Ga^
3		0.00 ^Bc^	20.68 ± 0.07 ^Bb^	20.84 ± 0.02 ^Ba^		29.01 ± 0.12 ^Dc^	30.41 ± 0.09 ^Db^	46.80 ± 0.13 ^Da^
4		93.60 ± 0.07 ^Ac^	160.01 ± 0.17 ^Ab^	299.10 ± 0.17 ^Aa^		414.44 ± 0.09 ^Ac^	504.37 ± 0.07 ^Ab^	868.61 ± 0.08 ^Aa^
5		0.00 ^Ba^	0.00 ^Ea^	0.00 ^Ea^		0.00 ^G^c	15.03 ± 0.12 ^Fb^	18.84 ± 0.19 ^Fa^
6		0.00 ^Bc^	3.01 ± 0.04 ^Db^	4.06 ± 0.06 ^Da^		11.01 ± 0.05 ^Ec^	15.51 ± 0.11 ^Eb^	29.35 ± 0.13 ^Ea^
7		0.00 ^Ba^	0.00 ^Ec^	0.00 ^Eb^		36.91 ± 0.06 ^Cc^	65.33 ± 0.16 ^Cb^	115.25 ± 0.14 ^Ca^
8		0.00 ^Ba^	0.00 ^Eb^	0.00 ^Ec^		409.13 ± 0.12 ^Bc^	453.70 ± 0.47 ^Bb^	478.69 ± 0.38 ^Ba^
	2‐octanone				2‐nonanone			
1	992.92	0.00 ^Ab^	0.00 ^Ab^	3.55 ± 0.12 ^Ba^	1092.32	0.00 ^Da^	0.00 ^Da^	0.00 ^Fa^
2		0.00 ^Ab^	0.00 ^Ab^	0.00 ^Ca^		0.00 ^Da^	0.00 ^Da^	1.99 ± 0.15 ^Ea^
3		0.00 ^Aa^	0.00 ^Aa^	0.00 ^Ca^		0.00 ^Da^	0.00 ^Da^	0.00 ^Fa^
4		0.00 ^Ab^	0.00 _Ab_	0.00 ^Ca^		162.75 ± 0.12 ^Ac^	170.72 ± 0.11 ^Ab^	189.35 ± 0.08 ^Ba^
5		0.00 ^Ab^	0.00 ^Ab^	12.16 ± 0.07 ^Aa^		0.00 ^Db^	0.00 ^Db^	5.24 ± 0.13 ^Da^
6		0.00 ^Aa^	0.00 ^Aa^	0.00 ^Ca^		0.00 ^Da^	0.00 ^Da^	0.00 ^Fa^
7		0.00 ^Aa^	0.00 ^Aa^	0.00 ^Ca^		9.37 ± 0.11 ^Cc^	13.46 ± 0.13 ^Cb^	24.93 ± 0.12 ^Ca^
8		0.00 ^Aa^	0.00 ^Aa^	0.00 ^Ca^		133.06 ± 0.07 ^Bc^	151.27 ± 0.12 ^Bb^	277.58 ± 0.06 ^Aa^
	2‐decanone				2‐undecanone			
1	1155.34	0.00 ^Aa^	0.00 ^Aa^	0.00 ^Ca^	1293.77	0.00 ^Ca^	0.00 ^Da^	0.00 ^Ca^
2		0.00 ^Aa^	0.00 ^Aa^	0.00 ^Ca^		0.00 ^Ca^	0.00 ^Da^	0.00 ^Ca^
3		0.00 ^Aa^	0.00 ^Aa^	0.00 ^Ca^		0.00 ^Ca^	0.00 ^Da^	0.00 ^Ca^
4		0.00 ^Ab^	0.00 ^Ab^	3.81 ± 0.07 ^Aa^		26.29 ± 0.06 ^Bc^	27.51 ± 0.02 ^Bb^	28.54 ± 0.09 ^Ba^
5		0.00 ^Ab^	0.00 ^Aa^	0.00 ^Ca^		0.00 ^Cb^	3.21 ± 0.11 ^Ca^	0.00 ^Cb^
6		0.00 ^Aa^	0.00 ^Aa^	0.00 ^Ca^		0.00 ^Ca^	0.00 ^Da^	0.00 ^Ca^
7		0.00 ^Aa^	0.00 ^Aa^	000 ^Ca^		0.00 ^Ca^	0.00 ^Da^	0.00 ^Ca^
8		0.00 ^Ab^	0.00 ^Ab^	3.19 ± 0.07 ^Ba^		32.57 ± 0.03 ^Ac^	33.47 ± 0.05 ^Ab^	72.46 ± 0.05 ^Aa^

*Note*: ^A–H^Different uppercase letters within a column and ketone indicate significant differences between butter and butter oil samples according to Duncan's multiple‐range test (*p* < .05). ^a–c^Different lowercase letters within a row (sample) indicate significant differences between storage periods according to Duncan's multiple‐range test (*p* < .05).

As with aldehydes, the highest total ketone values were obtained from butter oil samples produced at 120°C, and ketone values increased depending on the storage time (Figure 2). Storage time, production temperature, and raw material differences had a significant impact (*p* < .05) on all ketone quantities. When milk fat is heated in the presence of water, it is reported to form a homologous series of ketones (Lee et al., 1991; Van Der Ven et al., 1963).

Gundogdu et al. (2020) reported fluctuating changes in ketone values in butter samples. They detected 11 ketones (diacetyl, 2‐pentanone, 2‐hexanone, 2‐cyclohexanone, 2‐heptanone, 2‐octanone, 2‐nonanone, 2‐decanone, 2‐undecanone, 2‐dodecanone, 2‐tridecanone, and 2‐pentadecanone) in butter samples heated at different temperatures (100, 150, or 200°C), and it was noted that both aldehydes and ketones significantly increased with an increase in temperature. Tahmas‐Kahyaoğlu et al. (2022) detected the following ketones in butter samples produced from cow, sheep, and goat milk: 2‐propanone, 2‐butanone, 3‐methyl‐2‐pentanone, diacetyl, 2‐pentanone, 2‐heptanone, 2‐octanone, acetoin, 2‐nonanone, and 2‐undecanone. Even in small amounts, ketones can contribute to typical aromas such as fruity and floral (Vagenas & Ioannis, 2012).

Ethanol, 1‐pentanol, 1‐butanol, 3‐methyl‐1‐butanol, 1‐octanol, and 1‐hexanol are volatile alcohols detected in butter and butter oil samples (Table 6). The total content of volatile alcohols in all samples increased over time (Figure 2). Ethanol, which is the most dominant volatile alcohol and was found only in butter oil samples on days 1 and 30 of storage, was detected in all samples by the end of the storage period. In butter samples produced from yogurt and cream using different cultures, it has been reported that ethanol values varied between 1.9 and 48.9 on the 60th day of storage (Gundogdu et al., 2020). The ethanol values of butter samples in our study are consistent with these values.

**TABLE 6:** Volatile alcohol values (μg/100 g) of butter and butter oil samples (values are means ± SD, *n* = 3).

Sample no	Storage time (days)							
	RI	0	30	60	RI	0	30	60
	Ethanol				1‐Butanol			
1	464.60	0.00 ^Gb^	0.00 ^Gb^	2.98 ± 0.06 ^Ga^	660.13	0.00 ^Aa^	0.00 ^Da^	0.00 ^Ea^
2		4.07 ± 0.06 ^Ec^	8.15 ± 0.08 ^Fb^	19.90 ± 0.08 ^Ca^		0.00 ^Ab^	0.00 ^Db^	0.44 ± 0.04 ^Da^
3		10.05 ± 0.08 ^Ac^	12.85 ± 0.08 ^Db^	13.93 ± 0.08 ^Fa^		0.00 ^Ab^	0.00 ^Db^	0.39 ± 0.06 ^Da^
4		9.21 ± 0.06 ^Bc^	11.94 ± 0.09 ^Db^	16.61 ± 0.06 ^Ea^		0.00 ^Ac^	1.80 ± 0.09 ^Ab^	2.27 ± 0.06 ^Aa^
5		0.00 ^Gb^	0.00 ^Gb^	16.51 ± 0.06 ^Ea^		0.00 ^Aa^	0.00 ^Da^	0.00 ^Ea^
6		8.00 ± 0.08 ^Cc^	14.91 ± 0.04 ^Bb^	25.40 ± 0.08 ^Ba^		0.00 ^Aa^	0.00 ^Da^	0.00 ^Ea^
7		2.80 ± 0.12 ^Fc^	36.82 ± 0.11 ^Ab^	74.13 ± 0.08 ^Aa^		0.00 ^Ac^	0.62 ± 0.08 ^Cb^	1.03 ± 0.06 ^Ca^
8		6.32 ± 0.08 ^Dc^	13.15 ± 0.07 ^Cb^	19.42 ± 0.07 ^Da^		0.00 ^Ac^	1.13 ± 0.09 ^B^	1.81 ± 0.07 ^Bab^
	3‐methyl‐1‐butanol				1‐pentanol			
1	732.51	0.00 ^Bb^	0.00 ^Eb^	1.00 ± 0.06 ^Ea^	766.33	0.00 ^Ba^	0.00 ^Da^	0.00 ^Fa^
2		0.00 ^Bb^	0.00 ^Eb^	0.51 ± 0.02 ^Fa^		0.00 ^Ba^	0.00 ^Da^	0.00 ^Fa^
3		0.00 ^Bb^	0.00 ^Eb^	0.55 ± 0.05 ^Fa^		0.00 ^Bb^	0.00 ^Db^	3.18 ± 0.07 ^Da^
4		0.00 ^Ba^	0.00 ^Ea^	0.00 ^Ga^		0.00 ^Bc^	4.13 ± 0.06 ^Ab^	5.13 ± 0.06 ^Ba^
5		2.02 ± 0.05 ^Ac^	10.72 ± 0.09 ^Ab^	32.74 ± 0.09 ^Aa^		0.00 ^Ba^	0.00 ^Da^	0.00 ^Fa^
6		0.00 ^Bc^	5.61 ± 0.04 ^Bb^	7.61 ± 0.08 ^Ba^		0.00 ^Bc^	2.19 ± 0.07 ^Cb^	4.95 ± 0.07 ^Ca^
7		0.00 ^Bc^	1.90 ± 0.03 ^Cb^	4.99 ± 0.09 ^Ca^		0.00 ^Bb^	0.00 ^Db^	1.70 ± 0.07 ^Ea^
8		0.00 ^Bc^	1.38 ± 0.06 ^Db^	1.70 ± 0.07 ^Da^		3.43 ± 0.06 ^Ab^	3.49 ± 0.06 ^Bb^	6.96 ± 0.07 ^Aa^
	1‐hexanol				1‐octanol			
1	869.97	0.00 ^Ca^	0.00 ^Ca^	0.00 ^Da^	1005.91	0.00 ^Ca^	0.00 ^Ca^	0.00 ^Ca^
2		0.00 ^Ca^	0.00 ^Ca^	0.00 ^Da^		0.00 ^Ca^	0.00 ^Ca^	0.00 ^Ca^
3		0.00 ^Ca^	0.00 ^Ca^	0.00 ^Da^		0.00 ^Ca^	0.00 ^Ca^	0.00 ^Ca^
4		2.96 ± 0.08 ^Ac^	3.72 ± 0.09 ^Ab^	5.12 ± 0.10 ^Aa^		5.79 ± 0.04 ^Ac^	6.22 ± 0.05 ^Ab^	7.32 ± 0.36 ^Ba^
5		0.00 ^Ca^	0.00 ^Ca^	0.00 ^Da^		0.00 ^Ca^	0.00 ^Ca^	0.00 ^Ca^
6		0.00 ^Ca^	0.00 ^Ca^	0.00 ^Da^		0.00 ^Ca^	0.00 ^Ca^	0.00 ^Ca^
7		0.00 ^Cb^	0.00 ^Db^	0.90 ± 0.07 ^Ca^		0.00 ^Ca^	0.00 ^Ca^	0.00 ^Ca^
8		1.33 ± 0.05 ^Bc^	2.61 ± 0.05 ^Bb^	4.30 ± 0.06 ^Ba^		5.02 ± 0.09 ^Bc^	6.08 ± 0.07 ^Bb^	8.88 ± 0.06 ^Aa^

*Note*: ^A–G^Different uppercase letters within a column and alcohol indicate significant differences between butter and butter oil samples according to Duncan's multiple‐range test (*p* < .05). ^a–c^Different lowercase letters within a row (sample) indicate significant differences between storage periods according to Duncan's multiple‐range test (*p* < .05).

Lactic acid bacteria, especially heterofermentative ones (especially *Leuconostoc, Oenococcus*, and some *Lactobacillus* species), produce some ethanol (Lindsay, 1996, Ruiz‐Rodríguez et al., 2017). Therefore, it can be said that the starter cultures used in our study (*L. lactis* subsp. *lactis* bv. *diacetylactis* and *Leu. mesenteroides* subsp. *cremoris*) produce a certain amount of ethanol. However, the raw milk microflora has produced a higher amount of alcohol. Alcohol can be formed through various mechanisms. For instance, they can be formed as a result of the metabolism of lactic acid bacteria or as secondary products of free radical–induced lipid oxidation. Additionally, methyl alcohols can result from the catabolism of branched‐chain amino acids, while primary alcohols can be formed from the catabolism of aldehydes and methyl ketones (Centeno et al., 2003; Morales et al., 2003).

1‐Pentanol, 1‐butanol, 1‐octanol, and 1‐hexanol were not detected in the butter samples during storage. However, in butter oil samples, the highest values of these volatile alcohols were obtained from samples processed at 120°C. The values of 3‐methyl‐1‐butanol showed fluctuating changes, and the highest values in both groups were obtained from the butter samples. While the alcohol values of butter samples were significantly influenced by the use of cultures, the volatile alcohol values of butter oil were significantly affected by the raw material, production temperature, and storage duration (*p* < .05).

In samples of butter and butter oil, six acids have been identified, namely acetic, butanoic, pentanoic, hexanoic, heptanoic, and octanoic acids. Among these acids, the most dominant ones are butanoic and hexanoic acids. In the study conducted by Erfani et al. (2020), they reported that the main volatile acids found in ghee are butyric acid and hexanoic acid. Butanoic and hexanoic acids were detected in all samples throughout the storage period, and it was found that both acid values were statistically significantly higher in butter than in ghee at the end of storage (*p* < .05). Similar results were also found for octanoic acid at the end of storage. Pentanoic and heptanoic acids were detected in butter samples during the 30^th^ and 60^th^ days of storage, while none of the butter oil samples contained these fatty acids. Pentanoic and heptanoic acids are compounds formed as a result of enzymatic and microbial activity. The former has a cheesy and rancid odor, while the latter has an unpleasant odor (Moio et al., 2000).

At the beginning of storage, acetic acid was only detected in butter oil processed from uncultured butter, and at the end of storage, it was found in all samples. The acetic acid content in butter oil samples was higher than in butter samples at the end of storage. Acetic acid is one of the primary fermentation products of heterofermentative lactic acid bacteria (e.g., *Leu. citrovorum*) and contributes to the characteristic aroma of butter (Lindsay, 1996).

Apart from acetic acid, the amount of volatile acids was higher in butter samples than in butter oil samples. The quantities of all acids detected in the samples increased over time. The use of cultures, production temperatures, and storage duration have shown a significant effect (*p* < .05) on the acid values of the samples. In butter oil, enzymatic and chemical hydrolysis occurs at lower levels compared to butter. This is because ghee has lower water activity compared to butter, and the enzymatic activity is limited due to the heat treatment applied during production. Therefore, the acid levels of butter oil were lower than those of butter (Gundogdu et al., 2020; Nawar, 1996; Sserunjogi et al., 1998). It is known that volatile acids, especially the ones with short and medium chains, significantly contribute to the flavor of butter and butter oil (Yadav & Srinivasan, 1985).

Six hydrocarbons have been identified in samples of butter and butter oil (Table 7), and their amount has increased over time. In both groups, the lowest hydrocarbon values were generally detected in butter oil samples produced at 120°C. Hydroperoxides break down in a few steps yielding a wide variety of decomposition products such as aldehydes and hydrocarbons. Generally, cleavage on the carboxyl side results in the formation of an aldehyde and an acid, while cleavage on the methyl side produces a hydrocarbon and an oxoacid. These decomposition products can undergo further oxidation and degradation (Nawar, 1996). Erfani et al. (2020) identified dodecane, tetradecane, hexadecane, and octadecane in Iranian ghee, while Wadodkar et al. (2002) detected pentane, octane, toluene, and undecane in different types of oils. Hydrocarbons, which are secondary products of lipid autoxidation, do not directly affect the aroma, but they contribute to the formation of other aroma compounds (Bintsis & Robinson, 2004).

**TABLE 7:** Volatile acid and hydrocarbon values (μg/100 g) of butter and butter oil samples (values are means ± SD, *n* = 3).

Sample no	Storage time (days)							
	RI	0	30	60	RI	0	30	60
	Acids				Hydrocarbons			
	Acetic acid				Hexane			
1	576.55	0.00 ^Db^	0.00 ^Gb^	2.83 ± 0.15 ^Ha^	600	3.98 ± 0.09 ^Ec^	11.94 ± 0.09 ^Eb^	35.55 ± 0.08 ^Da^
2		0.00 ^Dc^	5.29 ± 0.12 ^Fa^	15.23 ± 0.08 ^Ea^		7.56 ± 0.10 ^Cc^	12.03 ± 0.06 ^Eb^	24.14 ± 0.11 ^Fa^
3		0.00 ^Dc^	6.26 ± 0.06 ^Db^	11.36 ± 0.12 ^Ga^		11.51 ± 0.011 ^Bc^	30.64 ± 0.07 ^Bb^	54.00 ± 0.12 ^Ba^
4		0.00 ^Dc^	5.32 ± 0.07 ^Fb^	19.17 ± 0.09 ^Da^		0.00 ^Gb^	0.00 ^Fb^	4.77 ± 0.08 ^Ga^
5		0.00 ^Dc^	5.84 ± 0.06 ^Eb^	12.07 ± 0.12 ^Fa^		2.87 ± 0.09 ^Fc^	31.57 ± 0.04 ^Ab^	58.89 ± 0.06 ^Aa^
6		13.43 ± 0.11 ^Bc^	27.73 ± 0.10 ^Cb^	30.02 ± 0.09 ^Ca^		12.32 ± 0.12 ^Ac^	13.88 ± 0.08 ^Db^	52.55 ± 0.08 ^Ca^
7		8.65 ± 0.12 ^Cc^	31.23 ± 0.09 ^Ab^	40.02 ± 0.08 ^Ba^		6.57 ± 0.64 ^Dc^	15.81 ± 0.04 ^Cb^	35.19 ± 0.09 ^Ea^
8		16.85 ± 0.13 ^Ac^	29.83 ± 0.16 ^Bb^	111.93 ± 0.10 ^Aa^		0.00 ^Ga^	0.00 ^Fa^	0.00 ^Ha^
	Butanoic acid				Heptane			
1	794.63	71.45 ± 0.11 ^Hc^	305.33 ± 0.09 ^Ab^	308.56 ± 0.06 ^Ba^	700	2.41 ± 0.06 ^Ac^	4.75 ± 0.09 ^Cb^	18.59 ± 0.08 ^Ca^
2		77.62 ± 0.02 ^Fc^	78.91 ± 0.17 ^Gb^	80.78 ± 0.06 ^Ga^		0.00 ^Dc^	2.55 ± 0.11 ^Db^	17.85 ± 0.06 ^Da^
3		96.76 ± 0.12 ^Dc^	104.85 ± 0.16 ^Eb^	118.88 ± 0.05 ^Ea^		0.00 ^Dc^	22.78 ± 0.07 ^Ab^	24.21 ± 0.08 ^Aa^
4		72.15 ± 0.06 ^Gc^	74.37 ± 0.11 ^Hb^	79.39 ± 0.38 ^Ha^		0.00 ^Db^	0.00 ^Eb^	3.73 ± 0.04 ^Ga^
5		130.55 ± 0.04 ^Ac^	219.77 ± 0.13 ^Bb^	445.78 ± 0.10 ^Aa^		1.39 ± 0.04 ^Cc^	14.36 ± 0.13 ^Bb^	19.72 ± 0.11 ^Ba^
6		80.08 ± 0.06 ^Ec^	86.67 ± 0.10 ^Fb^	115.24 ± 0.09 ^Fa^		2.01 ± 0.05 ^Bc^	4.81 ± 0.06 ^Cb^	15.76 ± 0.06 ^Ea^
7		98.30 ± 0.06 ^Cc^	125.60 ± 0.09 ^Db^	132.02 ± 0.10 ^Da^		0.00 ^Db^	0.00 ^Eb^	14.71 ± 0.06 ^Fa^
8		120.75 ± 0.15 ^Bc^	132.37 ± 0.06 ^Cb^	134.34 ± 0.06 ^Ca^		0.00 ^Da^	0.00 ^Ea^	0.00 ^Ha^
	Pentanoic acid				Benzene methyl			
1	885.86	0.00 ^Ac^	5.84 ± 0.08 ^Bb^	9.13 ± 0.06 ^Aa^	762.63	13.09 ± 0.08 ^Dc^	28.29 ± 0.08 ^Eb^	71.30 ± 0.09 ^Da^
2		0.00 ^Aa^	0.00 ^Ca^	0.00 ^Ba^		32.51 ± 0.08 ^Ac^	35.46 ± 0.07 ^Cb^	83.86 ± 0.06 ^Ca^
3		0.00 ^Aa^	0.00 ^Ca^	0.00 ^Ba^		25.23 ± 0.09 ^Bc^	59.66 ± 0.06 ^Ab^	98.12 ± 0.09 ^Ba^
4		0.00 ^Aa^	0.00 ^Ca^	0.00 ^Ba^		0.00 ^Gc^	2.66 ± 0.07 ^Hb^	25.98 ± 0.06 ^Ga^
5		0.00 ^Ab^	6.93 ± 0.08 ^Aa^	0.00 ^Bb^		9.43 ± 0.08 ^Ec^	53.58 ± 0.07 ^Bb^	60.62 ± 0.07 ^Fa^
6		0.00 ^Aa^	0.00 ^Ca^	0.00 ^Ba^		22.54 ± 0.10 ^Cc^	31.09 ± 0.06 ^Db^	60.64 ± 0.06 ^Fa^
7		0.00 ^Aa^	0.00 ^Ca^	0.00 ^Ba^		13.20 ± 0.05 ^Dc^	28.12 ± 0.10 ^Fb^	100.65 ± 0.06 ^Aa^
8		0.00 ^Aa^	0.00 ^Ca^	0.00 ^Ba^		5.61 ± 0.08 ^Fc^	6.66 ± 0.09 ^Gb^	61.56 ± 0.07 ^Ea^
	Hexanoic acid				Octane			
1	987.28	53.39 ± 0.08 ^Dc^	366.47 ± 0.09 ^Ab^	570.83 ± 0.07 ^Aa^	800	0.00 ^Ac^	54.60 ± 0.08 ^Ab^	112.56 ± 0.09 ^Aa^
2		52.12 ± 0.11 ^Ec^	54.13 ± 0.06 ^Gb^	61.11 ± 0.07 ^Fa^		0.00 ^Ac^	8.66 ± 0.06 ^Db^	37.48 ± 0.05 ^Da^
3		47.67 ± 0.06 ^Gc^	56.12 ± 0.03 ^Fb^	56.48 ± 0.08 ^Ga^		0.00 ^Ab^	36.28 ± 0.08 ^Ca^	36.42 ± 0.09 ^Ea^
4		45.25 ± 0.08 ^Hc^	51.02 ± 0.07 ^Hb^	52.11 ± 0.04 ^Ha^		0.00 ^Ab^	0.00 ^Eb^	7.51 ± 0.06 ^Fa^
5		79.22 ± 0.11 ^Bc^	130.22 ± 0.09 ^Bb^	250.55 ± 0.19 ^Ba^		0.00 ^Ac^	47.27 ± 0.08 ^Bb^	75.10 ± 0.11 ^Ba^
6		63.55 ± 0.07 ^Cc^	71.16 ± 0.07 ^Eb^	75.72 ± 0.11 ^Ea^		0.00 ^Ab^	0.00 ^Eb^	45.06 ± 0.03 ^Ca^
7		48.67 ± 0.06 ^Fc^	75.03 ± 0.06 ^Db^	129.49 ± 0.06 ^Da^		0.00 ^Ab^	0.00 ^Eb^	5.50 ± 0.13 ^Ga^
8		89.00 ± 0.15 ^Ac^	98.43 ± 0.05 ^Cb^	156.08 ± 0.07 ^Ca^		0.00 ^Aa^	0.00 ^Ea^	0.00 ^Ha^
	Heptanoic acid				Decane			
1	1077.77	0.00 ^Ab^	0.00 ^Ab^	6.10 ± 0.05 ^Aa^	1000	0.00 ^Ab^	0.00 ^Eb^	6.84 ± 0.07 ^Ga^
2		0.00 ^Aa^	0.00 ^Aa^	0.00 ^Ca^		0.00 ^Ab^	0.00 ^Eb^	5.84 ± 0.04 ^Ha^
3		0.00 ^Aa^	0.00 ^Aa^	0.00 ^Ca^		0.00 ^Ac^	8.47 ± 0.07 ^Ab^	11.73 ± 0.08 ^Ea^
4		0.00 ^Aa^	0.00 ^Aa^	0.00 ^Ca^		0.00 ^Ac^	6.91 ± 0.06 ^Bb^	9.02 ± 0.11 ^Fa^
5		0.00 ^Ab^	0.00 ^Ab^	1.33 ± 0.04 ^Ba^		0.00 ^Ab^	0.00 ^Eb^	53.11 ± 0.12 ^Aa^
6		0.00 ^Aa^	0.00 ^Aa^	0.00 ^Ca^		0.00 ^Ac^	5.98 ± 0.05 ^Cb^	18.82 ± 0.10 ^Ba^
7		0.00 ^Aa^	0.00 ^Aa^	0.00 ^Ca^		0.00 ^Ac^	4.09 ± 0.06 ^Db^	15.65 ± 0.07 ^Ca^
8		0.00 ^Aa^	0.00 ^Aa^	0.00 ^Ca^		0.00 ^Ab^	0.00 ^Eb^	13.51 ± 0.06 ^Da^
	Octanoic acid				2‐methylundecane			
1	1174.58	6.63 ± 0.08 ^Dc^	120.75 ± 0.08 ^Ab^	173.27 ± 0.08 ^Aa^	1148.55	0.00 ^Ab^	0.00 ^Ab^	2.43 ± 0.04 ^Da^
2		5.11 ± 0.05 ^Ec^	8.01 ± 0.07 ^Fb^	9.27 ± 0.03 ^Fa^		0.00 ^Aa^	0.00 ^Aa^	0.00 ^Ea^
3		4.02 ± 0.09 ^Gc^	4.41 ± 0.04 ^Gb^	5.01 ± 0.14 ^Ga^		0.00 ^Ab^	0.00 ^Ab^	2.74 ± 0.10 ^Ca^
4		4.24 ± 0.05 ^Fc^	4.49 ± 0.06 ^Gb^	4.68 ± 0.07 ^Ha^		0.00 ^Aa^	0.00 ^Aa^	0.00 ^Ea^
5		14.70 ± .06 ^Ac^	115.82 ± 0.08 ^Bb^	118.42 ± 0.10 ^Ba^		0.00 ^Ab^	0.00 ^Ab^	4.45 ± 0.06 ^Aa^
6		9.11 ± 0.04 ^Cc^	9.51 ± 0.09 ^Eb^	10.42 ± 0.07 ^Ea^		0.00 ^Aa^	0.00 ^Aa^	0.00 ^Ea^
7		3.86 ± 0.05 ^Hc^	10.30 ± 0.05 ^Db^	14.77 ± 0.08 ^Da^		0.00 ^Ab^	0.00 ^Ab^	2.88 ± 0.08 ^Ba^
8		12.77 ± 0.08 ^Bb^	14.04 ± 0.07 ^Ca^	2.20 ± 0.09 ^Cc^		0.00 ^Aa^	0.00 ^Aa^	0.00 Ea

*Note*: ^A–H^Different uppercase letters within a column and acid and hydrocarbon indicate significant differences between butter and butter oil samples according to Duncan's multiple‐range test (*p* < .05). ^a–c^Different lowercase letters within a row (sample) indicate significant differences between storage periods according to Duncan's multiple‐range test (*p* < .05).

Alpha‐pinene, D‐limonene, sabinene, and beta‐myrcene are terpenes identified in samples of butter and butter oil, and their amounts showed fluctuating variations in the samples (Table 8). Among the five terpenes detected in the samples, D‐limonene was the most predominant. The values of sabinene exhibited irregular changes during the storage period, but the values at the end of storage were generally significantly higher than the initial values. At the beginning of the study, beta‐myrcene was not detected in all samples. However, after this stage, it was detected on the 30th and 60th days of storage period in butter and butter oil samples. Li et al. (2012) reported the detection of one terpen in yak butter. Gundogdu et al. (2020) stated that they found three terpenes in butter samples produced from yogurt and cream. Terpenes are secondary metabolites found in plants, and their quantities in milk can vary depending on various factors such as geographical region, season, and the variety of plants in the feed (Bontinis et al., 2012).

**TABLE 8:** Volatile terpene (α‐pinene, D‐limonene, sabinene, β‐myrcene, and myrcene) and ester (*butanoic acid*, *methyl ester*, *butanoic acid*, 2‐*methyl‐, methyl ester,* and *hexanoic acid*, *methyl ester*) values (μg/100 g) of butter and butter oil samples (values are means ± SD, *n* = 3).

Sample no	Storage time (days)							
	RI	0	30	60	RI	0	30	60
	α‐Pinene				*Butanoic acid, methyl ester*			
1	930.94	0.00 ^Ac^	5.90 ± 0.05 ^Bb^	11.67 ± 0.08 ^Ea^	721.44	0.00 ^Ac^	0.93 ± 0.06 ^Ab^	1.33 ± 0.11 ^Ba^
2		0.00 ^Ab^	0.00 ^Cb^	7.01 ± 0.05 ^Ga^		0.00 ^Aa^	0.00 ^Ca^	0.00 ^Ca^
3		0.00 ^Ab^	0.00 ^Cb^	35.28 ± 0.06 ^Ba^		0.00 ^Aa^	0.00 ^Ca^	0.00 ^Ca^
4		0.00 ^Aa^	0.00 ^Ca^	0.00 ^Ha^		0.00 ^Aa^	0.00 ^Ca^	0.00 ^Ca^
5		0.00 ^Ac^	26.43 ± 0.05 ^Ab^	95.74 ± 0.06 ^Aa^		0.00 ^Ac^	0.53 ± 0.04 ^Bb^	5.65 ± 09 ^Aa^
6		0.00 ^Ab^	0.00 ^Cb^	12.15 ± 0.07 ^Da^		0.00 ^Aa^	0.00 ^Ca^	0.00 ^Ca^
7		0.00 ^Ab^	0.00 ^Cb^	10.23 ± 0.06 ^Fa^		0.00 ^Aa^	0.00 ^Ca^	0.00 ^Ca^
8		0.00 ^Ab^	0.00 ^Cb^	29. 63 ± 0.06 ^Ca^		0.00 ^Aa^	0.00 ^Ca^	0.00 ^Ca^
	Sabinene				*Butanoic acid*, 2‐*methyl‐, methyl ester*			
1	973.75	4.05 ± 0.06 ^Ac^	4.39 ± 0.06 ^Bb^	4.90 ± 0.05 ^Da^	767.02	0.00 ^Ac^	2.33 ± 0.04 ^Ab^	4.08 ± 0.05 ^Ba^
2		0.00 ^Da^	3.27 ± 0.07 ^Cb^	3.57 ± 0.07 ^Ea^		0.00 ^Aa^	0.00 ^Ca^	0.00 ^Ca^
3		0.00 ^Dc^	4.51 ± 0.12 ^Bb^	12.22 ± 0.08 ^Ba^		0.00 ^Aa^	0.00 ^Ca^	0.00 ^Ca^
4		0.00 ^Dc^	1.75 ± 0.06 ^Fb^	1.90 ± 0.05 ^Ga^		0.00 ^Aa^	0.00 ^Ca^	0.00 ^Ca^
5		1.78 ± 0.08 ^Bc^	10.32 ± 0.07 ^Ab^	28.26 ± 0.06 ^Aa^		0.00 ^Ac^	1.79 ± 0.08 ^Bb^	14.78 ± 0.05 ^Aa^
6		0.00 ^Db^	1.92 ± 0.07 ^Ea^	1.97 ± 0.07 ^Ga^		0.00 ^Aa^	0.00 ^Ca^	0.00 ^Ca^
7		0.00 ^Fc^	1.95 ± 0.04 ^Eb^	2.58 ± 0.09 ^Fa^		0.00 ^Aa^	0.00 ^Ca^	0.00 ^Ca^
8		1.41 ± 0.05 ^Cc^	2.18 ± 0.08 ^Db^	8.42 ± 0.08 ^Ca^		0.00 ^Aa^	0.00 ^Ca^	0.00 ^Ca^
	β‐Myrcene				*Hexanoic acid*, *methyl ester*			
1	990.37	0.00 ^Aa^	0.00 ^Da^	0.00 ^Ea^	925.41	0.00 ^Ac^	2.20 ± 0.03 ^Ab^	4.07 ± 0.08 ^Ba^
2		0.00 ^Ac^	1.88 ± 0.10 ^Bb^	3.45 ± 0.13 ^Aa^		0.00 ^Aa^	0.00 ^Ca^	0.00 ^Ca^
3		0.00 ^Ab^	0.00 ^Db^	1.53 ± 0.04 ^Ca^		0.00 ^Aa^	0.00 ^Ca^	0.00 ^Ca^
4		0.00 ^Aa^	0.00 ^Da^	0.00 ^Ea^		0.00 ^Aa^	0.00 ^Ca^	0.00 ^Ca^
5		0.00 ^Aa^	0.00 ^Da^	0.00 ^Ea^		0.00 ^Ac^	1.82 ± 0.10 ^Bb^	14.83 ± 0.11 ^Aa^
6		0.00 ^Ac^	1.05 ± 0.08 ^Bb^	2.21 ± 0.07 ^Ba^		0.00 ^Aa^	0.00 ^Ca^	0.00 ^Ca^
7		0.00 ^Ac^	0.45 ± 0.11 ^Cb^	1.22 ± 0.09 ^Da^		0.00 ^Aa^	0.00 ^Ca^	0.00 ^Ca^
8		0.00 ^Aa^	0.00 ^Da^	0.00 ^Ea^		0.00 ^Aa^	0.00 ^Ca^	0.00 ^Ca^
	D‐Limonene							
1	1026.94	8.50 ± 0.06 ^Fc^	25.23 ± 0.10 ^Fb^	81.42 ± 0.08 ^Ca^				
2		6.35 ± 0.07 ^Gc^	48.03 ± 0.12 ^Cb^	49.45 ± 0.12 ^Fa^				
3		13.44 ± 0.08 ^Cc^	55.57 ± 0.12 ^Ab^	101.82 ± 0.06 ^Aa^				
4		18.70 ± 0.06 ^Ac^	28.73 ± 0.10 ^Db^	39.93 ± 0.06 ^Ga^				
5		11.02 ± 0.09 ^Dc^	50.50 ± 0.07 ^Bb^	52.02 ± 0.08 ^Ea^				
6		17.87 ± 0.05 ^Bc^	19.62 ± 0.10 ^Hb^	75.62 ± 0.07 ^Da^				
7		10.60 ± 0.07 ^Ec^	20.92 ± 0.11 ^Gb^	75.62 ± 0.07 ^Da^				
8		0.00 ^Hc^	25.80 ± 0.08 ^Eb^	85.58 ± 0.58 ^Ba^				

*Note*: A‐H Different uppercase letters within a column and terpene and ester indicate significant differences between butter and butter oil samples according to Duncan's multiple‐range test (*p* < .05). a‐c Different lowercase letters within a row (sample) indicate significant differences between storage periods according to Duncan's multiple‐range test (*p* < .05).

During the storage period, no ester group was found on butter oil samples. However, in the samples of butter, three ester compounds were detected on the 30th and 60th days of storage: butanoic acid methyl ester, butanoic acid, and 2‐methyl ester heptanoic acid. The quantities of all three compounds significantly increased (*p* < .05) depending on the storage period (Table 8). Wadodkar et al. (2002) reported the presence of four esters, namely dimethyl phthalate, diethyl phthalate, isobutyl phthalate, and dibutyl phthalate, in samples of butter and butter oil. On the other hand, Gündoğdu et al. (2020) reported that they detected 10 esters in butter. Esters are cleavage products of hydroperoxides. Cleavage on the acid side of the hydroperoxides results in formation of an aldehyde and an acid or ester. Fruity aroma‐imparting esters contribute positively to the overall flavor balance in dairy products such as cheese and butter at low concentrations. However, at high concentrations, they can lead to flavor defects (Liu et al., 2004).

## 4 CONCLUSIONS

According to the results of the study, there is no significant difference in fatty acid composition and atherogenicity index between butter and butter oil samples. However, based on our previous studies, it can be said that butter oil has significantly lower water activity which may confer some advantages, compared to butter. These advantages are related to microbial and chemical stability. Free fatty acid, total volatile aldehyde, ketone, acid, and alcohol values of all samples increase during storage. The highest increases in free fatty acid values occurred in butter, and the highest increases in total volatile aldehyde, ketone, acid, and alcohol values occurred in ghee samples processed at 120°C. The study results have shown that butter oil has a lower content of free fatty acids compared to butter, which is crucial in preventing aroma defects and oxidation in butter oil. However, the aldehyde and ketone values of butter oil produced at 120°C are significantly higher than the aldehyde and ketone values of butter oil produced at 60°C and 90°C and butter samples. These results indicate that the heat treatment applied at 120°C may pose a risk of thermal oxidation in butter oil. Thermal oxidation can result in off‐flavors, rancidity, and a decrease in nutritional value in the butter oil, making it less desirable for consumption.

The results indicate that processing butter into butter oil at an appropriate temperature can enhance the oxidative stability of butter oil. Considering that butter oil is often produced from unpasteurized butter in small‐scale operations and households, it can be concluded that applying heat treatment at 90°C can provide both microbiological and chemical stability to the product without compromising properties.

## AUTHOR CONTRIBUTIONS

**Tekin Demir:** Data curation (equal); investigation (equal); methodology (equal); resources (equal); validation (equal). **Seval Andiç:** Conceptualization (lead); data curation (equal); funding acquisition (lead); methodology (equal); project administration (lead); supervision (lead); writing – original draft (equal). **Şehriban Oğuz:** Methodology (equal); resources (equal); validation (equal); writing – original draft (equal).

## FUNDING INFORMATION

This research was funded by the Scientific Research Projects Coordination Unit of Van Yuzuncu Yil University (Van, Turkey) under number FDK‐2017‐6331.

## CONFLICT OF INTEREST STATEMENT

The authors declare that they have no known competing financial interests or personal relationships that could have appeared to influence the work reported in this paper.

## Data Availability

Data are available on request.
